# Characteristics of Quinolone Resistance in *Escherichia coli* Isolates from Humans, Animals, and the Environment in the Czech Republic

**DOI:** 10.3389/fmicb.2016.02147

**Published:** 2017-01-09

**Authors:** Magdalena Röderova, Dana Halova, Ivo Papousek, Monika Dolejska, Martina Masarikova, Vojtech Hanulik, Vendula Pudova, Petr Broz, Miroslava Htoutou-Sedlakova, Pavel Sauer, Jan Bardon, Alois Cizek, Milan Kolar, Ivan Literak

**Affiliations:** ^1^Department of Microbiology, Faculty of Medicine and Dentistry, Palacký University OlomoucOlomouc, Czechia; ^2^Department of Biology and Wildlife Diseases, Faculty of Veterinary Hygiene and Ecology, University of Veterinary and Pharmaceutical Sciences BrnoBrno, Czechia; ^3^Central European Institute of Technology (CEITEC), University of Veterinary and Pharmaceutical Sciences BrnoBrno, Czechia; ^4^Department of Infectious Diseases and Microbiology, Faculty of Veterinary Medicine, University of Veterinary and Pharmaceutical Sciences BrnoBrno, Czechia; ^5^Department of Microbiology, University Hospital OlomoucOlomouc, Czechia; ^6^Institute of Applied Biotechnologies (IAB)Prague, Czechia

**Keywords:** *Escherichia coli*, quinolone resistance, human, animals, wastewater, MLST, PFGE

## Abstract

*Escherichia coli* is a common commensal bacterial species of humans and animals that may become a troublesome pathogen causing serious diseases. The aim of this study was to characterize the quinolone resistance phenotypes and genotypes in *E. coli* isolates of different origin from one area of the Czech Republic. *E. coli* isolates were obtained from hospitalized patients and outpatients, chicken farms, retailed turkeys, rooks wintering in the area, and wastewaters. Susceptibility of the isolates grown on the MacConkey agar with ciprofloxacin (0.05 mg/L) to 23 antimicrobial agents was determined. The presence of plasmid-mediated quinolone resistance (PMQR) and ESBL genes was tested by PCR and sequencing. Specific mutations in *gyrA, gyrB, parC*, and *parE* were also examined. Multilocus sequence typing and pulsed-field gel electrophoresis were performed to assess the clonal relationship. In total, 1050 *E. coli* isolates were obtained, including 303 isolates from humans, 156 from chickens, 105 from turkeys, 114 from the rooks, and 372 from wastewater samples. PMQR genes were detected in 262 (25%) isolates. The highest occurrence was observed in isolates from retailed turkey (49% of the isolates were positive) and inpatients (32%). The *qnrS1* gene was the most common PMQR determinant identified in 146 (56%) followed by *aac(6*′*)-Ib-cr* in 77 (29%), *qnrB19* in 41 (16%), and *qnrB1* in 9 (3%) isolates. All isolates with high level of ciprofloxacin resistance (>32 mg/L) carried double or triple mutations in *gyrA* combined with single or double mutations in *parC*. The most frequently identified substitutions were Ser(83)Leu; Asp(87)Asn in GyrA, together with Ser(80)Ile, or Glu(84)Val in ParC. Majority of these isolates showed resistance to beta-lactams and multiresistance phenotype was found in 95% isolates. Forty-eight different sequence types among 144 isolates analyzed were found, including five major clones ST131 (26), ST355 (19), ST48 (13), ST95 (10), and ST10 (5). No isolates sharing 100% relatedness and originating from different areas were identified. In conclusion, our study identified PMQR genes in *E. coli* isolates in all areas studied, including highly virulent multiresistant clones such as ST131 producing CTX-M-15 beta-lactamases.

## Introduction

*Escherichia coli* is a common part of the gastrointestinal flora of humans and animals but some strains can cause serious diseases. Quinolones are the antimicrobial agents of choice for treatment of various infections caused by *E. coli* or other Gram-negative bacteria. Because of extensive use for multiple clinical indications in human or veterinary medicine, bacterial resistance to quinolones has developed over the time (Andriole, 2005). The World Health Organization indicated fluoroquinolones as critically important drugs for human medicine due to a strong correlation observed between the consumption and an increase in the resistance, and they recommended prudent use of fluoroquinolones both in human and veterinary medicine (World Health Organization, 2012).

According to the European Antimicrobial Resistance Surveillance Network (EARS-Net), an interactive database, an alarming emergence of fluoroquinolone-resistant invasive *E. coli* isolates is evident in European countries (European Centre for Disease Prevention and Control, 2015). Quinolone antibiotics are one of the most widely used antimicrobial agents in the treatment of urinary tract infections. Unfortunately, the extensive use has led to the increase of the rate of *E. coli* isolates resistant to fluoroquinolones all over the world (Lautenbach et al., 2004; Dalhoff, 2012). Resistance to nalidixic acid and ciprofloxacin was reported as high or extremely high in isolates from broilers in European countries (ECDC, EFSA, and EMA, 2015). *E. coli* isolates resistant to fluoroquinolones has been described in food-producing and companion animals (Platell et al., 2011; Gosling et al., 2012; Literak et al., 2013; Agabou et al., 2016) as well as in wild animals and the environment (Jiménez Gómez et al., 2004; Colomer-Lluch et al., 2013).

Target sites for quinolones in *E. coli* strains are the bacterial topoisomerases, namely DNA gyrase (topoisomerase II) as the primary site and topoisomerase IV as a secondary target. Both enzymes are essential for bacterial DNA replication. Mutations in specific domains of *gyrA, gyrB, parC*, and *parE* cause single aminoacid changes in either gyrase or topoisomerase IV that contribute to quinolone resistance. Multiple mutations in the quinolone-determining resistant regions (QRDR) of topoisomerase enzymes are usually associated with high-level of fluoroquinolone resistance in *E. coli* strains (Hopkins et al., 2005). Target-mediated resistance represents the most common and clinically most significant form (Ruiz, 2003; Aldred et al., 2014).

The first identified plasmid-mediated quinolone resistance (PMQR) gene was discovered in 1998 and termed *qnrA1* (Martínez-Martínez et al., 1998). So far, three families of plasmid-mediated mechanisms associated with quinolone resistance have been identified: i/Qnr proteins protecting target enzymes DNA gyrase and topoisomerase IV from quinolone inhibition ii/ aminoglycoside acetyltransferase Aac(6′)-Ib-cr acetylating several fluoroquinolones such as ciprofloxacin and norfloxacin and iii/ efflux pumps QepA and OqxAB removing antibiotics from bacterial cells. PMQR provide only a low level of quinolone resistance, not reaching the clinical breakpoints defined by the Clinical and Laboratory Standard Institute criteria (CLSI, 2015). However, PMQR genes may facilitate the selection of higher-level resistance in the presence of quinolones and lead to treatment failure (Strahilevitz et al., 2009; Jacoby et al., 2014).

The aims of this study were to determine the occurrence of PMQR in *E. coli* isolates from humans, food-producing animals, wild animals and wastewater samples from one defined area in the Czech Republic and to compare genetic characteristics of PMQR-positive *E. coli* isolates from various sources as well as to outline possible ways of their transmission between humans and animals.

## Material and methods

### Sample collection

Between May 2013 and December 2014, *E. coli* isolates were obtained from human clinical materials (urine, stool, sputum, blood, bile, endotracheal aspirate, samples from cannulas, and bronchoalveolar lavages) of patients hospitalized at the University Hospital Olomouc (UHO) in the Czech Republic and from urine samples from outpatients in the community of Olomouc Region. The community subjects had neither been hospitalized in the previous 3 months nor had been living in nursing homes.

In the same period (May 2013 and December 2014), *E. coli* isolates were obtained from environmental samples from chicken farms (*n* = 2628) and cloacal swabs from market-weight turkeys at slaughterhouses (*n* = 120) in the eastern part of the Czech Republic (Olomouc and South Moravian Region). Environmental samples were taken from bedding using gauze shoe covers worn by a worker who walked through a poultry house. Turkey cloacal swabs were collected at slaughterhouses and placed into Amies transport medium.

Wastewaters were taken during 2013–2015 in six sampling sites that included wastewater treatment plants (WWTP) from four hospitals located in three towns of the Olomouc Region (Olomouc, Prostejov and Sternberk) (WWTP1-4), the abattoir in Prerov (WWTP5) and WWTP near Henčlov (WWTP6). In total, 124 wastewater samples were examined including 21 samples from WWTP1 (in 2013-6 samples; 2014-13; 2015-2), 25 from WWTP2 (2014-20, 2015-5), 19 from WWTP3 (in 2013-6; 2014-11, 2015-2), 19 from WWTP4 (in 2013-6; 2014-11, 2015-2), 21 from WWTP5 (collected weekly for a period of 4 months in 2013/2014, plus 3 control samplings were made in May and August 2014) and 19 from WWTP6 (collected weekly for a period of 4 months in 2013/2014). Cellulose swabs were immersed into wastewater at the inflow and/or outflow of sampling points for 48 h according to the procedure described (Moore et al., 1952).

Faecal rook (*Corvus frugilegus*) samples (*n* = 595) were collected from roosting places used by rook flocks in the Olomouc Region. The samples were collected in Prerov in October, November and December 2012, in Tovacov from January till March 2013, and in Troubky in November 2013. The sampling method has been described previously (Literak et al., 2012). Smears from fresh feces were taken by cotton swabs tampons and inserted into Amies transport medium.

### Selective cultivation of *E. coli* and detection of PMQR genes

After delivery to the laboratory, collected samples were handled according to the following procedures in order to isolate *E. coli*.

Samples from the environment of chicken farms were placed into peptone water and incubated aerobically for 24 h at 37°C. Subsequently, the peptone water was inoculated onto MacConkey agar with ciprofloxacin (0.05 mg/L; MCA_CIP_) and cultivated overnight. Swabs from retailed turkeys were subcultivated directly on MCA_CIP_ overnight. One isolate per sample was selected for further phenotypic and genetic analysis.

Swabs taken from wastewaters were inserted into a sterile bottle with peptone water and incubated at 37°C for 24 h. The enriched samples of peptone water were subcultivated on MCA_CIP_ overnight. One to ten colonies of lactose-positive colonies showing different morphology were taken from each MCA_CIP_ and subjected to further examination.

Faecal swabs from rooks were incubated in buffered peptone water at 37°C overnight and subsequently cultivated on MCA_CIP_ overnight. In this type of sample, also only one isolate per sample was selected.

Human clinical material was handled according to the types of material and cultivated aerobically for 24 h at 37°C. A total number of 3521 *E. coli* isolates were collected and from these, three hundred and three isolates were randomly selected for detection of PMQR genes. The collection analyzed included 270 resistant (MIC of ciprofloxacin >0.5 mg/L) and 33 sensitive (MIC of ciprofloxacin ≤ 0.5 mg/L) isolates according to the European Committee in Antimicrobial Susceptibility Testing (EUCAST) criteria.

The species identification of all isolates was confirmed using matrix-assisted laser desorption/ionization-time of flight mass spectrometer (MALDI-TOF MS) (Biotyper Microflex, Bruker Daltonik GmbH, Bremen, Germany).

Genomic DNA of all *E. coli* isolates obtained using heat lysis was used as a template for PCR detection of PMQR genes (*aac(6*′*)-Ib-cr, qepA, qnrA, qnrB, qnrC, qnrD, qnrS, oqxAB*), followed by sequencing of the amplicons. As positive controls, well-known characterized strains were included in each reaction (Table S1).

### Antimicrobial susceptibility testing of PMQR-positive isolates

For each PMQR-positive *E. coli* isolate, susceptibilities to ampicillin (the ranges of tested concentrations for the antimicrobial substance were 0.5–64 mg/L, ampicillin/sulbactam (0.5–64), cefazolin (0.5–64), cefuroxime (0.5–64), gentamicin (0.25–32 or 0.5–64), trimethoprim/sulfamethoxazole (1–128), colistin (0.25–32), oxolinic acid (0.25–32 or 0.5–64), ofloxacin (0.125–16 or 0.25–32), tetracycline (0.25–32), aztreonam (1–64), piperacillin (2–256), piperacillin/tazobactam (2–256), cefoperazone (0.25–32), cefotaxime (0.125–16), ceftazidime (0.125–16), cefepime (0.125–16), cefoperazone/sulbactam (0.5–64), meropenem (0.125–16), ciprofloxacin (0.125–16 or 0.25–32), tigecycline (0.06–8), tobramycin (0.25–32), and amikacin (0.5–32) were tested using the standard microdilution method according to the EUCAST (European Committee on Antimicrobial Susceptibility Testing) breakpoint criteria (European Centre for Disease Prevention and Control, 2015). Microdilution antibiotic panels were prepared using dispensing instrument DYNAMIC 3000 automated system (DYNEX, Czech Republic). *E. coli* ATCC 25922 and *E. coli* ATCC 35218 were used as reference strains for quality control.

### Detection of ESBL genes in PMQR-positive isolates

All PMQR-positive isolates of *E. coli* with the minimum inhibitory concentration (MIC) of the tested 3rd and 4th generation cephalosporins ≥1 mg/L were screened for extended-spectrum beta-lactamase production (ESBL) using Jarlier's double-disk synergy test (DDST) which was modified by including a disk with cefepime and another disk with ceftazidime and ceftazidime/clavulanic acid (Jarlier et al., 1988; Htoutou Sedlakova et al., 2011). Each ESBL-producing isolate was screened for *bla*_CTX-*M*,_ *bla*_TEM_ and *bla*_SHV_ genes by PCR followed by sequencing of amplicons of genes responsible for ESBL phenotype (Table S1).

### Detection of mutations in the topoisomerase genes and MLST analysis of PMQR-positive isolates

From a group of 262 PMQR-positive *E. coli* isolates, 144 isolates from all of the studied areas were selected for further characterization that included the detection of specific mutations in *gyrA, gyrB, parC* and *parE* genes and multilocus sequence typing (MLST). Isolates were selected randomly in order to cover all the studied areas.

Total genomic DNA from *E. coli* strains was prepared from an overnight culture (16 h, 37°C) grown on meat-peptone agar using DNeasy Blood & Tissue kit (QIAGEN, Germany) according to the manufacturer's recommendations.

PCR amplification of the part of *gyrA, gyrB, parC* and *parE* genes that included the sequence of QRDRs was performed using previously described primers (Oram and Fisher, 1991; Vila et al., 1994, 1996; Ruiz et al., 1997). The reaction mixture contained complete reaction buffer with MgCl_2_ (containing 100 mmol/L Tris-HCl (pH 8.8), 500 mmol/L KCl, 1% Triton X-100, 15 mmol/L MgCl_2_ (Top-Bio, Czech Republic), 0.5 U of *Taq* DNA polymerase (Top-Bio), 0.4 μmol/L primer concentration for each primer, 40 μmol/L concentration of deoxynucleoside triphosphates and 1 μL of template DNA. The PCR was run in the Light Cycler96 instrument (Roche, USA) under the following conditions: initial denaturation at 95°C for min, 30 cycles of denaturation (95°C for 30 s), annealing (58°C for 30 s), extension (72°C for 60 s), and final extension at 72°C for 7 min.

MLST of seven housekeeping genes (*adk, fumC, gyrB, icd, mdh, purA, recA*) was performed according to the MLST protocol standardized for *E. coli* (http://mlst.warwick.ac.uk/mlst/).

For each of the 144 samples, 11 amplicons (4 topoisomerase and 7 MLST amplicons) were pooled in an equimolar ratio to generate one single sample and sent to the laboratories of the Institute of Applied Biotechnologies (Prague, Czech Republic) for sequencing. DNA libraries were constructed using the Nextera XT DNA Sample Preparation Kit (Illumina, Inc., San Diego, CA) and two multiplex sequencing assays (1 × 96 samples, 1 × 48 samples) were performed on the Illumina MiSeq platform (Illumina, Inc. San Diego, CA).

### NGS data analysis

A quality control check of pair-end FASTQ files was performed as a first step. Bases with Phred Quality Score lower than defined threshold (threshold = 30) were filtered out. Quality control included check for possible adapter contamination. Then all variants of reference sequences of single housekeeping genes of *E. coli* (*adk, fumC, gyrB, icd, mdh, purA, recA*) in FASTA file were downloaded (http://mlst.warwick.ac.uk/mlst/dbs/Ecoli) and indexed by Burrows-Wheeler Aligner (BWA version 0.7.13; Li and Durbin, 2010). Paired-end FASTQ files were aligned against all indexed reference FASTA files using BWA-mem algorithm with minimum seed length = 19, matching score = 1, mismatch penalty = 4, gap open penalty = 6, and gap extension penalty = 1. Generated Sequence Alignment Map (SAM format) was converted into its binary form (BAM format) and sorted by coordinates. Unmapped sequences reads were filtered out from BAM files. Algorithms Samtools (samtools version 0.1.18) and VarScan (Koboldt et al., 2009) were applied for variant calling using the following parameters: minimum coverage = 10, minimum mapping quality = 10, minimum reads = 2, minimum variant frequency = 0.05, and *p* = 0.05 were used for variant calling. Generated Variant Calling Format (VCF format) was statistically analyzed and a minimum number of variants (required 0, this number means that our FASTQ files have 100% match with a variant of the reference gene) was evaluated like a gene form of selected housekeeping gene of *E. coli*. All bioinformatics analyses were performed on Operating System Linux (Ubuntu 14.04 LTS) and using programming language Python.

Single point mutations in selected part of *gyrA, gyrB, parC* and *parE* including QRDRs were evaluated using the Integrative Genomics Viewer IGV version 2.3. (Broad Institute, Cambrige, UK) (https://www.broadinstitute.org/software/igv). The genome sequence *E. coli* MG1655 (NC_000913.2) was selected as a reference sequence for alignment.

Sanger sequencing was performed with 11 samples to confirm the results of MLST analysis carried out by NGS. This was done because some problems occurred in the assignment of the appropriate allele in MLST analysis. Gene sequences were subsequently submitted to the *E. coli* MLST Database, University of Warwick (http://mlst.warwick.ac.uk/mlst/dbs/Ecoli/). Obtained MLST allele sequences were compared with the results generated by NGS.

### Pulsed-field gel electrophoresis

*E. coli* isolates belonging to the same sequence type (ST) according to MLST analysis and originating from different sources (human, animal or wastewater) were subjected to examination of their epidemiological relationship using pulsed-field gel electrophoresis (PFGE). The total genomic DNA was isolated from overnight bacterial culture according to the procedure published (Husickova et al., 2012). All samples were digested with *Xba*I (30 U for 3 h) (Takara, Bio, Otsu, Shiga, Japan) and subjected to PFGE. The resulting restriction profiles were analyzed with the GelCompar II software (Applied Maths, Kortrijk, Belgium) using the Dice coefficient (1,5%) for comparing similarity and unweighted pair group method using arithmetic averages for cluster analysis. The results were interpreted according to criteria described by Tenover et al. (1995).

## Results

### PMQR genes in *E. coli* isolates with reduced susceptibility or resistance to ciprofloxacin

A collection of 1050 isolates of *E. coli* with reduced susceptibility or resistance to ciprofloxacin was obtained from humans, food producing, or wild animals and wastewater samples from the area of the Olomouc and South Moravian Regions, Czech Republic (Table 1). The highest occurrence of *E. coli* strains isolated from MCA_*CIP*_ of animal originwas detected from turkey samples (105 isolates per 120 samples), the lowest from chicken samples (156 isolates per 2628 samples). In human population, 303 *E. coli* isolates were selected according to the procedure described in material and methods section. From the total amount of 124 wastewater samples, 372 *E. coli* isolates were analyzed.

**Table 1:** **Distribution and characterization of PMQR-positive isolates of *E. coli* from different areas of collection**.

**Origin**	**No. of collected samples**	**No. of collected isolates**	**No. of PMQR-positive isolates**	***qnrB1***	***qnrB4***	***qnrB8***	***qnrB10***	***qnrB19***	***qnrD*1**	***qnrS1***	***qnrS2***	***aac(6′)-Ib-cr***	***oqxAB***	**No. of isolates subjected to NGS**
Hospital	18954	220	70 (31.8%)	7	1	–	–	2	–	6	–	60	1	33
Community	nd	83	14 (16.9%)	1	1	–	–	–	–	4	–	9	–	9
Chicken	2628	156	17 (10.9%)	–	–	1	–	4	–	12	–	–	1	10
Turkey	120	105	51 (48.6%)	–	–	–	–	16	–	46	–	–	–	50
Rook	595	114	20 (17.5%)	–	–	–	–	5	2	9	–	4	–	11
Wastewater	124	372	90 (24.2%)	1	–	–	1	14	–	69	4	4	–	31
Total		1050	262 (25.0%)	9	2	1	1	41	2	146	4	77	2	144

*nd, data for community samples not available*.

*From a total number of 124 wastewater samples 26 contained at least one PMQR-positive isolate of E. coli*.

PMQR genes were identified in 262 (25%) *E. coli* isolates (Table 1). The gene *qnrS1* was the most prevalent, found in 56% of PMQR-positive isolates from all studied areas. It was identified in 76% PMQR-positive isolates from poultry farms, turkey, rooks and wastewater. The second most common gene was *aac(6*′*)-Ib-cr* found in 82% of human PMQR-positive clinical isolates followed by *qnrB19* that dominated in isolates from turkey and wastewater samples. The *qnrA, qnrC, and qepA* genes were not found.

Twenty-three isolates carried two different PMQR genes of the following combination: *qnrB19*+*qnrS1* (12 isolates), *qnrB1*+*aac(6*′*)-Ib-cr* (7), *qnrB4*+*aac(6*′*)-Ib-cr* (2), *qnrS1*+*oqxAB* (1) *qnrB10*+*qnrS1* (1).

In case of wastewaters where more than one *E. coli* colony per sample was examined, the occurrence of several *E. coli* isolates carrying different PMQR genes and originating the same water sample was detected. Six wastewater samples contained *E. coli* isolates carrying *qnrS1* together with *E. coli* carrying *qnrB19* were detected. In one sample *E. coli* harboring *qnrB1* and *E. coli* with *qnrS1* were found. The last sample contained two different types of *E. coli*, one carrying *aac(6*′*)-Ib-cr* and the other with *qnrS1*.

### Antimicrobial susceptibility of PMQR-positive isolates

For each PMQR-positive *E. coli* isolate, susceptibility to 23 antimicrobial agents was tested (Table S2). Isolates from hospitalized patients showed the highest level of resistance including ampicillin (97%), piperacillin (96%), third generation cephalosporins (63% for cefotaxime and 43% for ceftazidime), tetracycline (83%), and tobramycin (86%). Isolates from the community displayed high level of resistance to ampicillin (93%), tetracycline (93%), piperacillin (93%), tobramycin (64%), and resistance to third generation cephalosporins (50.0% for cefotaxime and 43% for ceftazidime). On the other hand, wastewater isolates were less resistant to tested antibiotics. High level of resistance was detected only for ampicillin (79%), tetracycline (83%) and piperacillin (78%).

Resistance to ampicillin was common also among isolates from retailed turkeys (96%), chicken farms (71%) and rook feces (75%). Other frequent resistance phenotypes included tetracycline and piperacillin found in 77 and 59% of chicken, in 86 and 90% from turkey and in 75 and 70% from rook isolates, respectively. The majority of *E. coli* isolates from food-producing animals were susceptible to third generation cephalosporins while 20% of rook isolates showed resistance. Most isolates from the inpatients and the community showed resistance to ciprofloxacin. A total of 270 isolates were resistant (MIC of ciprofloxacin >0.5 mg/L) and 33 sensitive (MIC of ciprofloxacin ≤ 0.5 mg/L) according to the EUCAST criteria. However, this results is influenced by different selection criteria for human isolates since isolates with MIC of ciprofloxacin >0.5 mg/L were preferably included in the study. In contrast, only 50 of all PMQR-positive isolates from other studied areas showed resistance to ciprofloxacin with the highest level observed in isolates from chicken farms (41%). A total of 36% of animal *E. coli* isolates (chicken, turkey, and rook) were resistant to oxolinic acid, 32% to ofloxacin and 28% to ciprofloxacin. In the group of wastewater samples, 28% of isolates displayed resistance to oxolinic acid as well as to ciprofloxacin, and 34% to ofloxacin. The distribution of MIC values of tested quinolones in selected isolates (*n* = 144) and the comparison with the results of genetic detection is summarized in the Table 2.

**Table 2:** **Distribution of substitutions in topoisomerase subunits, PMQR determinants and MIC of tested quinolones in *E. coli* isolates**.

**Substitutionsin topoisomerase subunits**				**PMQR**	**No. of isolates**	**MIC range (mg/L)**			**Origin of isolates**
**GyrA**	**GyrB**	**ParC**	**ParE**			**OXO**	**OFL**	**CIP**	
Ser(83)Leu	−	−	−	*qnrB1*	2	16–>32	0.5–2	0.5–2	Hospital, community
Ser(83)Leu	−	−	−	*qnrB19*	3	64	2	0.5	Wastewater
Ser(83)Leu	−	−	−	*qnrS1*	4	16–64	0.25–8	<0.125–4	Wastewater, hospital, chicken
Ser(83)Leu	−	Ser(80)Arg	Ser(458)Pro	*qnrS1*	1	>32	16	16	Chicken
Ser(83)Leu; Asp(87)Asn	−	Ser(80)Arg	Ser(458)Pro	*qnrS1*	1	>64	16	>16	Turkey
Ser(83)Leu; Asp(87)Asn	−	Ser(80)Ile	Ser(458)Ala	*aac(6′)-Ib-cr*	3	>64	>16	>16	Rook
Ser(83)Leu; Asp(87)Asn	−	Ser(80)Ile	−	*qnrS1*	1	>128	2	8	Chicken
Ser(83)Leu; Asp(87)Asn	−	Ser(80)Ile^*^	Leu(445)His	*qnrS1*	1	>32	>16	>32	Hospital
Ser(83)Leu; Asp(87)Asn	−	Ser(80)Ile^*^	Ser(458)Ala	*aac(6′)-Ib-cr*	6	>32–>64	16–32	>16–>32	Hospital, community
Ser(83)Leu; Asp(87)Asn	−	Ser(80)Ile^*^	Ser(458)Ala	*qnrB1*	2	>64	>16	>32	Hospital
Ser(83)Leu; Asp(87)Asn	−	Ser(80)Ile^*^	Ser(458)Ala	*qnrS1*	1	>16	>32	>32	Hospital
Ser(83)Leu; Asp(87)Asn	−	Ser(80)Ile^*^	−	*qnrB19, qnrS1*	3	>64	8–32	8–>16	Chicken
Ser(83)Leu; Asp(87)Asn	−	Ser(80)Ile^*^	−	*qnrS1*	4	>32–>64	4–16	8–32	Community, chicken, turkey
Ser(83)Leu; Asp(87)Asn	−	Ser(80)Ile^*^; Glu(84)Val	Ser(458)Ala	*aac(6′)-Ib-cr*	1	>32	>32	>32	Hospital
Ser(83)Leu; Asp(87)Asn	−	Ser(80)Ile^*^; Ser(57)Thr	Leu(57)Phe	*qnrB1*	1	>32	>16	32	Hospital
Ser(83)Leu; Asp(87)Asn	−	Ser(80)Ile; Glu(84)Val	−	*aac(6′)-Ib-cr*	21	>32–>64	8–16	16–>32	Hospital, community, wastewater
Ser(83)Leu; Asp(87)Asn	−	Ser(80)Ile; Glu(84)Val	−	*qnrB1*	1	>32	16	>32	Hospital
Ser(83)Leu; Asp(87)Asn	−	−	−	*qnrS1*	1	>32	8	16	rook
Ser(83)Leu; Asp(87)Gly	−	Ser(80)Arg	−	*qnrS1*	1	>32	4	8	Chicken
Asp(87)Asn	−	−	−	*qnrS1*	1	16	2	2	Chicken
Ser(83)Leu; Ala(84)Val; Asp(87)Asn	−	Ser(80)Ile; Glu(84)Val	−	*aac(6′)-Ib-cr*	1	>32	8	32	Hospital
−	−	−	Arg(378)His	*qnrS1*	1	4	0,5	0.5	Chicken
−	−	−	Asp(475)Glu	*qnrB19, qnrS1*	1	8	0,5	0.5	Turkey
−	−	−	Asp(475)Glu	*qnrS1*	17	8–>32	<0.25–4	0.25–8	Turkey
−	−	−	−	*qnrB1*	2	<0.5–4	<0.125–0,25	<0.125–0.25	Hospital
−	−	−	−	*qnrB1, aac(6′)-Ib-cr*	1	2	0.5	0.5	Hospital
−	−	−	−	*qnrB19*	14	4–32	0.25–1	<0.125–0.5	Chicken, hospital, wastewater, rook
−	−	−	−	*qnrB19, qnrS1*	7	4–8	0.25–0.5	0.25–0.5	Turkey
−	−	−	−	*qnrD1*	1	8	0.5	0.125	Rook
−	−	−	−	*qnrS1*	39	1–32	0.25–4	0.125–4	wastewater, hospital, community, rook, chicken, Turkey
−	−	−	−	*qnrS1, qnrB10*	1	16	4	2	Wastewater

PMQR, plasmid-mediated quinolone resistance; OXO, oxolinic acid; OFL, ofloxacin; CIP, ciprofloxacin; MIC, minimum inhibitory concentration; nucleotide change ATT (marked as Ile) and ATC (marked as Ile^*^).

A total number of 66 isolates was screened for ESBLs using phenotypic and genetic methods. Production of ESBL enzymes was found in 61 *E. coli* isolates. Subsequent genetic analysis revealed the presence of *bla*_CTX-*M*-15_ in 51 isolates (hospital, community, wastewater). Two isolates from chicken and wastewater carried the gene *bla*_CTX-*M*-1_, *bla*_CTX-*M*-14_ was found in two isolates from hospital and wastewater and *bla*_SHV-12_ was detected in three isolates from wastewater. Three isolates showing ESBL phenotype were negative for all tested *bla* genes.

### Mutations in the topoisomerase genes in PMQR-positive isolates

Various mutations in *gyrA, parC* or *parE* genes were found in 60 out of 144 PMQR-positive isolates while no isolate with mutation in *gyrB* was observed (Table 2).

The most common GyrA substitution was Ser(83)Leu found in 59 isolates (41%), followed by Asp(87)Asn, detected in 49 isolates (34%). A total of 49 isolates carried double or triple substitution in GyrA.

Two aminoacid substitutions in ParC were found in 25 isolates (17%) with Ser(80)Ile and Glu(84)Val being the most common combination (*n* = 23). The most common substitution in ParE was Asp(475)Glu found in 18 isolates (13%).

All the *E. coli* isolates with a high level of ciprofloxacin resistance (>32 mg/L) were shown to carry double mutations in *gyrA* in combination with single or double mutations in *parC*. Substitutions at codon 83 and 87 in GyrA along with substitutions at codon 80 and 84 in *parC* gene were the most common. One isolate carried three mutations in *gyrA* with aminoacid changes at positions 83, 84, and 87. The majority of high-level ciprofloxacin resistant isolates were obtained from hospitalized patients and possessed the gene *aac(6*′*)-Ib-cr*. Other isolates with high level ciprofloxacin resistance originated from community and WWTP3 also carried *aac(6*′*)-Ib-cr*.

In most isolates susceptible to ciprofloxacin according to the EUCAST criteria (≤ 0.5 mg/L), no mutations in *gyrA, gyrB*, or *parC* were detected. However, five isolates possessed single aminoacid substitution at the codon 83 in GyrA (MIC of ciprofloxacin was 0.5 mg/L). All isolates were susceptible to almost all tested antimicrobial agents except ofloxacin (the MIC was 16–64 mg/L) and three of them were resistant to ofloxacin (MIC = 2 mg/L). Substitutions at position 378 [Arg(378)His)] or 475 [Asp(475) Glu] in ParE were found in 18 isolates susceptible to ciprofloxacin (MIC of ciprofloxacin was 0.2 or 0.5 mg/L). These strains displayed high level of resistance to ampicillin and piperacillin.

### Clonal relationship of PMQR-positive isolates

In the group of 144 isolates, analyzed by MLST, 48 different STs were identified (Table S3) including five major clones ST131 (*n* = 26), ST355 (19), ST48 (13), ST95 (10), and ST10 (5). Isolates of ST131 were obtained from various types of human clinical materials and from one wastewater sample, while the second most common clone ST355 carrying the *qnrS1* or the combination of *qnrS1*+*qnrB19* genes was found predominantly from retailed turkey. MLST analysis also showed the presence of identical STs in samples of different origin. Isolates of ST88 or ST226 were identified from chicken farms as well as from retailed turkeys. ST10, ST48, and ST95 were found in the poultry and wildlife (chicken farms, retailed turkey, and rooks) and in wastewater samples. Other sequence types identified in samples of different origin included ST428, ST533, and ST617. The determination of relevant ST failed in four samples. In these samples bioinformatics analysis did not assign appropriate allele variant and Sanger sequencing did not provide relevant sequences, suggesting the isolates belonged to atypical *E. coli* isolates untypable by MLST.

ST131 was most often associated with *bla*_CTX-*M*-15_ and *aac(6*′*)-Ib-cr*, however one strain harbored the combination of *bla*_CTX-*M*-15_ a *qnrB1*. The production of CTX-M-15 enzyme was also detected together with *qnrB1* in one ST393 and two ST410 strains. In three ST405 and one ST617 a combination of *bla*_CTX-*M*-15_ and *aac(6*′*)-Ib-cr* was identified. One ST48 *E. coli* isolate harbored *bla*_CTX-*M*-15_, together with *qnrB1* and *aac(6*′*)-Ib-cr*. In one *qnrS1*-postitive ST43 strain, *bla*_CTX-*M*-1_ gene was detected. Two *qnrS1*-positive and SHV-12 producing strains, one belonging to ST540 and the other to ST58 were identified. The other CTX-M-14 or CTX-M-15 producing strains were not analyzed using MLST.

**Figure 1:**
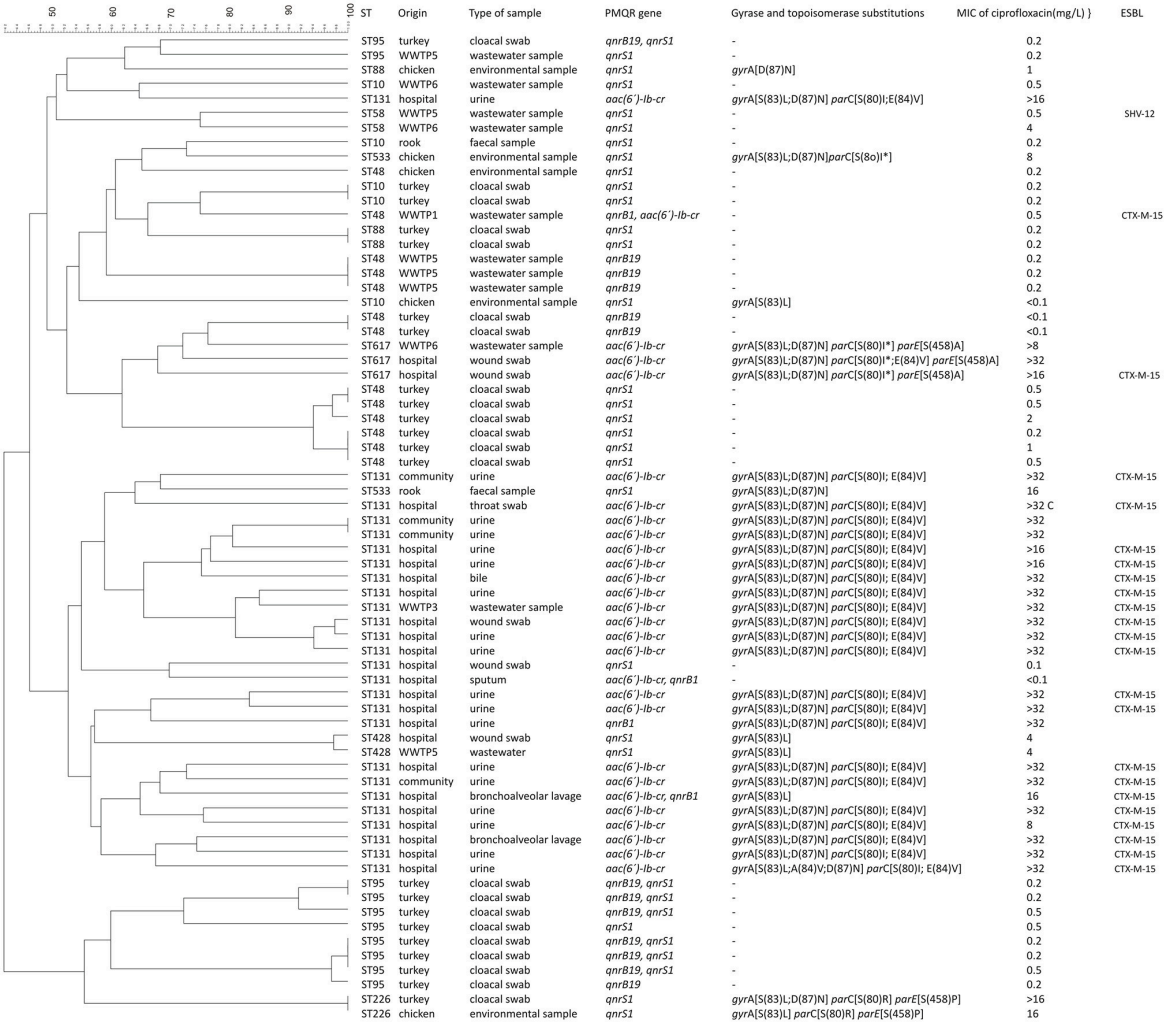
**Dendrogram of *E. coli* isolates including information about origin, PMQR genes, gyrase and topoisomerase substitutions, MIC of ciprofloxacin and beta-lactamases**.

*E. coli* isolates belonging to the same ST and originating from different sources (human, animal or environmental) were subjected to PFGE to determine the level of their genetic relatedness (Figure 1). Based on these criteria a total of 68 isolates of the following STs were analyzed: ST10 (*n* = 5), ST48 (*n* = 13), ST58 (*n* = 2), ST88 (*n* = 3), ST95 (*n* = 10), ST131 (*n* = 26), ST226 (*n* = 2), ST428 (*n* = 2), ST533 (*n* = 2), and ST617 (*n* = 3). Overall, a high diversity of restriction patterns was observed in isolates of the same ST. No isolates with 100% identity of PFGE profiles originating from different areas (human, animal, or environment) were identified.

Globally disseminated ST131 was predominant in samples from hospitalized patients as well as from community subjects while no isolates of this clonal group were found in food-producing animals or rooks. The majority (69%) of *E. coli* ST131 isolates collected from these samples displayed high-level of ciprofloxacin resistance (>32 mg/L), harbored double or triple mutations in *gyrA* along with double mutations in *parC* gene and the PMQR gene aac*(6*′*)-Ib-cr*. The overall similarity of PFGE patterns of ST131 isolates was <50% confirming high genetic diversity of this clonal group. Only two *E. coli* ST131 isolates originating from the same patient from which the samples were taken 17 days apart shared 100% identity.

Significant clonal similarity was detected only in isolates originating from single collection areas, including ST10, ST48, ST88, and ST95 from retailed turkeys. In the ST10 group two isolates shared 100% similarity of their PFGE profiles. Both isolates were susceptible to ciprofloxacin, carried *qnrS1*, and did not contain gyrase or topoisomerase mutations. A group of six closely related strains was detected in ST48 group. All of them were collected from turkeys, displayed susceptibility or a low level of resistance to ciprofloxacin (MIC 1 - 2 mg/L), harbored *qnrS1* and were originated from the same turkey farm. Another three ST48 isolates from WWTP5 displayed 100% identity of their PFGE patterns, originated from wastewater samples obtained from the same collection date and shared only 52% similarity of the PFGE patterns with ST48 isolates from turkey.

In the ST95 group, two and three strains from turkeys showed identical PFGE profiles. All isolates of the ST95 group were susceptible to ciprofloxacin, did not possess any mutations in gyrase or topoisomerase genes and most of them carried both *qnrB19* and *qnrS1* genes.

## Discussion

Quinolones are antimicrobial agents widely used in hospitals, community and veterinary practice. According to the combined data of ECDC, EFSA, EMA (ECDC, EFSA, and EMA, 2015), the consumption rate of fluoroquinolones reached 3.2 tons in primary care sector in the Czech Republic in 2012. The same data reported that 1.2 tons of fluoroquinolones were used in food producing animals in the country. However, the consumption of this class of antibiotics is still relatively low compared to other countries such as Germany, France, or Italy (ECDC, EFSA, and EMA, 2015).

The first aim of this study was to investigate the occurrence of *E. coli* isolates with PMQR genes in humans, food-producing animals, wild animals and wastewater samples from a defined area of the Czech Republic. The results pointed at 25% occurrence of *E*. *coli* with PMQR genes in all areas studied. The most common PMQR gene in *E. coli* from humans was *aac(6*′*)-Ib-cr* found mainly in ST131 clone with a high level of fluoroquinolone resistance. It has been demonstrated that *aac(6*′*)-Ib-cr* gene is more prevalent in *E. coli* ST131 than other PMQR genes (Cerquetti et al., 2009; Peirano et al., 2010; Hussain et al., 2012) and it is carried mainly by plasmids of incompatibility group F (IncF) along with genes encoding extended-spectrum beta-lactamase CTX-M or resistance to other antimicrobial classes (Woodford et al., 2009; Nicolas-Chanoine et al., 2014).

Regarding other types of PMQR determinants, *qnrS1* gene was identified in all areas examined. It was found (alone or in combination with *qnrB19*) in 73% of isolates that showed reduced susceptibility to ciprofloxacin and originated from wastewater samples or food-producing animals. Currently, this PMQR variant seems widespread in *E. coli* isolates from various types of food-producing animals in Europe (Forcella et al., 2010; Veldman et al., 2011). The second most common *qnr* variant, *qnrB19*, was found predominantly in the isolates from turkeys and wastewater samples. Both *qnrB19* and *qnrS1* were previously detected in chicken broilers in the Czech Republic (Literak et al., 2013). In *E. coli* isolates from rooks, *qnrB19, qnrD1, qnrS1* and *aac(6*′*)-Ib-cr* were identified. These PMQR variants have been previously detected in *E. coli* from rooks sampled in several European countries including the Czech Republic (Literak et al., 2012; Jamborova et al., 2015).

All *E. coli* isolates with high levels of ciprofloxacin resistance (MIC >32 mg/L), including ST131 clone, carried double or triple mutations in *gyrA* in combination with single or double mutations in *parC*. *E. coli* ST131 with specific substitutions in GyrA or ParC as found in our study have been previously described (Cerquetti et al., 2010; Platell et al., 2011; Paltansing et al., 2013). Isolates from poultry and rooks that displayed clinical levels of ciprofloxacin resistance linked to single or double mutations in *gyrA* and *parC* were found in our study. Strains of various animal origin with these types of mutations have been recently found worldwide (Sáenz et al., 2003; Liu et al., 2012; Castillo et al., 2013; Johnning et al., 2015; Balakrishnan et al., 2016).

ST131 was the predominant clone in our study found in the hospitalized patients as well as in the community subjects and also in one sample from the hospital WWTP. *E. coli* ST131 is a worldwide multidrug resistant clone responsible mainly for urinary but also for other types of infections and widely disseminated in hospital and community patients worldwide (Johnson et al., 2003; Cerquetti et al., 2010; Lee et al., 2010; Nicolas-Chanoine et al., 2014). In the Czech Republic, isolates of this ST producing CTX-M beta-lactamases were found in patients suffering from community or hospital acquired infections (Dolejska et al., 2012; Micenková et al., 2014; Papagiannitsis et al., 2015). Our results also pointed at the occurrence of CTX-M-15-producing ST131 clone in UHO. The ST131 clone has also been isolated from wildlife, companion animals, and retail chicken meat (Ewers et al., 2010; Ghodousi et al, 2016; Oh et al., 2016) as well as from wastewater or water samples (Dolejska et al., 2011a; Colomer-Lluch et al., 2013; Varela et al., 2015).

ST10 clonal complex (including ST10, ST34, ST43, ST48, ST167, and ST617) was the second most widespread in our study. It represents one of the largest clonal complexes within the *E. coli* MLST database, generally being antimicrobial-susceptible, with low virulence (Manges and Johnson, 2012) but it has also been associated with strains producing ESBL or AmpC beta-lactamases (Alcalá et al., 2016; Ben Said et al., 2016; Wang et al., 2016). Based on our results, one ST48 *E. coli* producing CTX-M-15 from wastewater, one ST617 strain with CTX-M-15 from wound swab and one ST43 *E. coli* with production of CTX-M-1 beta-lactamase from chicken were identified. In contrast to other studies (Huijbers et al., 2014; Agabou et al., 2016), no epidemiological link between human and animal isolates of this clonal group was observed in our study.

ST95 was the third largest clonal group in our study including isolates from cloacal swabs from market-weight turkeys and one isolate from a wastewater sample. This clone has been associated with infections in domestic (Mora et al., 2013) and wild birds (Jamborova et al., 2015) as well as urinary tract infections in humans (Riley, 2014). In contrast to ST131, ST95 lineage includes less multidrug resistant strains (Gibreel et al., 2012; Adams-Sapper et al., 2013). ST355 prevalent in our samples from market-weight turkey, seems to be not as widespread in the world as previous ones. It has been documented in *E*. *coli* isolates from urine and rectal samples in a multinational report by Adler et al. (2014) and in a study from the U.S. (Tartof et al., 2007).

Several authors suggested potential transmission of *E. coli* clones from animals to humans (Platell et al., 2011; Agabou et al., 2016; Nüesch-Inderbinen and Stephan, 2016) and the role of contaminated food in the local spread of resistant *E. coli* strains (Vincent et al., 2010). In a study from Platell et al. (2011), a high level of relatedness of *E. coli* ST131 strains from companion animals and humans was observed. Moreover, Agabou et al. (2016) demonstrated a clonal relationship between human and chicken ciprofloxacin-resistant *E. coli* isolates in North-Eastern Algeria. In contrast to these studies, we did not find any correlation between samples of human, animal, or environmental origin. No strains sharing 100% relatedness and originating from different areas of collection were identified using PFGE. However, some limitations of our study might affect the final results. These include the preferable selection of *E. coli* isolates resistant to ciprofloxacin from clinical materials of hospitalized patients and community subjects, the preferable selection of urine samples and the absence of rectal samples from a healthy population.

## Author contributions

Conception and design of the study: JB, AC, MK, IL. Sample collection: MR, DH, IP, MM, VH, VP, MH, PS, JB, and AC. Data analysis and interpretation, Final approval of the version to be published and drafting the article: MR, DH, IP, MD, MM, VH, VP, PB, MH, PS, JB, AC, MK, and IL. Critical revision of the article: MR, DH, IP, MD, MK, and IL. Agreement to be accountable for all aspects of the work in ensuring that questions related to the accuracy or integrity of any part of the work are appropriately investigated and resolved: MR, DH, IP, MD, MM, VH, VP, PB, MH, PS, JB, AC, MK, and IL.

## Funding

This work was supported by the grant Internal Grant Agency of Ministry of Health of the Czech Republic (NT/14398), Internal Grant Agency of the Palacký University Olomouc (IGA_LF_2016_022), Internal Grant Agency of the University of Veterinary and Pharmaceutical Sciences Brno, Brno, Czech Republic (217/2015/FVHE) and CEITEC 2020 - Central European Institute of Technology (CZ.1.05/1.1.00/02.0068) from European Regional Development Fund (LQ1601) from the Czech Ministry of Education, Youth and Sports within the National Programme for Sustainability II.

### Conflict of interest statement

The authors declare that the research was conducted in the absence of any commercial or financial relationships that could be construed as a potential conflict of interest.
