# Supplementary material for: Characteristics of Quinolone Resistance in Escherichia coli Isolates from Humans, Animals, and the Environment in the Czech Republic
Source: Front Microbiol. 2017 Jan 9;7:2147. doi: 10.3389/fmicb.2016.02147 (PMC5220107; doi:10.3389/fmicb.2016.02147)
Supplement: Supplementary file 1 [file Table1.docx]

**S1:** Primers for PMQR and beta-lactamase genes and PCR conditions used in this study

| **Primer** | **Sequence (5’-3’)** | **Target gene** | **Reference strains used as positive control** | **Annealing temperature (Ta, °C)** | **Reference** |
| --- | --- | --- | --- | --- | --- |
| aac(6’)Ib-F | TTG CGA TGC TCT ATG AGT GGC TA | *aac(6’)-Ib* | *E. coli* C1 with *aac(6’)-Ib-cr* | 55 | Park et al., 2006 |
| aac(6’)Ib-R | CTC GAA TGC CTG GCG TGT TT |  |  |  |  |
| qepA-F | TGG TCT ACG CCA TGG ACC TCA | *qepA* | *E. coli* C2 with *qepA* | 53 | Perichon et al., 2007; Yamane et al., 2007 |
| qepA-R | TGA ATT CGG ACA CCG TCT CCG |  |  |  |  |
| qnrA-F | ATT TCT CAC GCC AGG ATT TG | *qnrA* | *E. coli* C3 with *qnrA* | 53 | Robicsek et al., 2006 |
| qnrA-R | GAT CGG CAA AGG TTA GGT CA |  |  |  |  |
| qnrB-F* | GAT CGT GAA AGC CAG AAA GG | *qnrB* | *E. coli* C4 with *qnrB1* and *E. coli* CE1511 with *qnrB19* | 53 | Kim et al., 2009 |
| qnrB-R* | ATG AGC AAC GAT GCC TGG TA |  |  |  |  |
| qnrB_II† | CCA YAG CTC ACA YTT TTC SA |  |  | 53 | This study |
| qnrB_III† | ATG GCT CTG GCA CTC GTT |  |  |  |  |
| qnrB_VI† | Cta rcc aat may cgc gat gcc aag |  |  | 53 | Literak et al., 2012 |
| qnrB_VII† | ATG RCT CTG GCR TTA GTT RGC GAA |  |  |  |  |
| qnrB19.seq1F† | ATG ACT CTG GCA TTA GTT GG |  |  | 53 | Dolejska et al., 2011 |
| qnrB19.seq1R† | CCA CAG CTC ACA CTT TTC CA |  |  |  |  |
| qnrB19. seq2F† | TGC CAT TTT CAA AAG CTG TG |  |  | 53 | Dolejska et al., 2011 |
| qnrB19.seq2R† | GTA ACC AAT CAC AGC GAT GC |  |  |  |  |
| qnrB-1-10-F† | ATG ACG CCA TTA CTG TAT AAA AAA |  |  | 53 | Tamang et al., 2008 |
| qnrB-1-10-R† | CTA GCC AAT AAT CGC GAT GCC A |  |  |  |  |
| psp2† | AAA TTT AAY CAG AAA AAA GC |  |  | 55 | Jacoby et al., 2011 |
| sc3† | GCT SAR GAG AAC AGC TAT AC |  |  |  |  |
| ds2† | AAG AGT GGA AAA TTT CCA CA |  |  |  |  |
| ds3† | ATG GCT GAA GTT GAG ATT AT |  |  |  |  |
| qnrC-F | GGG TTG TAC ATT TAT TGA ATC G | *qnrC* | *E. coli* C5 with *qnrC* | 53 | Kim et al., 2009 |
| qnrC-R | CAC CTA CCC ATT TAT TTT CA |  |  |  |  |
| qnrD-F | CGA GAT CAA TTT ACG GGG AAT A | *qnrD* | *E. coli* C6 with *qnrD1* | 55 | Cavaco et al., 2009 |
| qnrD-R | AAC AAG CTG AAG CGC CTG |  |  |  |  |
| qnrS-F* | GCA AGT TCA TTG AAC AGG GT | *qnrS* | *E. coli* C7 with *qnrS1* | 53 | Cattoir et al., 2007 |
| qnrS-R* | TCT AAA CCG TCG AGT TCG GCG |  |  |  |  |
| qnrS1.seq1F† | ATG GAA ACC TAC AAT CAT ACA TAT CG |  |  | 55 | Dolejska et al., 2011b |
| qnrS1.seq1R† | TTC GTT CCT ATC CAG CGA TT |  |  |  |  |
| qnrS1.seq2F† | TTC GTG ATG CAA GTT TCC AA |  |  | 55 | Dolejska et al., 2011 |
| qnrS1.seq2R† | TTA GTC AGG ATA AAC AAC AAT ACC C |  |  |  |  |
| oqxA-F | CTC GGC GCG ATG ATG CT | *oqxA* | *E. coli* pOLA52 with *oqxAB* | 60 | Kim et al., 2009 |
| oqxA-R | CCA CTC TTC ACG GGA GAC GA |  |  |  |  |
| oqxBs | TTC TCC CCC GGC GGG AAG TAC | *oqxB* | *E. coli* pOLA52 with *oqxAB* | 64 | Kim et al., 2009 |
| oqxBa2 | CTC GGC CAT TTT GGC GCG TA |  |  |  |  |
| blaTEM-F | TT CTT GAA GAC GAA AGG GC | *bla*_TEM_ | *E. coli* NCTC 13351 with *bla*_TEM-3_ | 58 | Brinas et al., 2002 |
| blaTEM-R | ACG CTC AGT GGA ACG AAA AC |  |  |  |  |
| blaSHV-F | CAC TCA AGG ATG TAT TGT G | *bla*_SHV_ | *Klebsiella pneumoniae* NCTC 13368 with *bla*_SHV18_ | 58 | Brinas et al., 2002 |
| blaSHV-R | TTA GCG TTG CCA GTG CTC G |  |  |  |  |
| PANCTX-M-F* | TTT GCG ATG TGC AGT ACC AGT AA | *bla*_CTX-M_ (universal) | *E. coli* NCTC 13400 with *bla*_CTX-M-15_ | 58 | Lewis et al., 2007 |
| PANCTX-M-R* | CGA TAT CGT TGG TGG TGC CAT A |  |  |  |  |
| CTXM1-F3* | GAC GAT GTC ACT GGC TGA GC | *bla*_CTX-M-I group_ | *E. coli* NCTC 13400 with *bla*_CTX-M-15_ | 58 | Pitout et al., 2004 |
| CTXM1-R2* | AGC CGC CGA CGC TAA TAC A |  |  |  |  |
| CTX-M-1seq-F† | TCT TCC AGA ATA AGG AAT CCC | *bla*_CTX-M-I group_ | *E. coli* NCTC 13400 with *bla*_CTX-M-15_ | 58 | Dolejska et al., 2011 |
| CTX-M-1seq-R† | CCG TTT CCG CTA TTA CAA AC |  |  |  |  |
| CTXM914F* | GCT GGA GAA AAG CAG CGG AG | *bla*_CTX-M-IV group_ | *Enterobacter cloacae* NCTC13464 with *bla*_CTX-M-9 group_ | 58 | Pitout et al., 2004 |
| CTXM914R* | GTA AGC TGA CGC AAC GTC TG |  |  |  |  |
| CTX-M-9-like-F† | ATGGTGACAAAGAGAGTGCAACG | *bla*_CTX-M-IV group_ | *Enterobacter cloacae* NCTC13464 with *bla*_CTX-M-9 group_ | 58 | This study |
| CTX-M-9-like-R† | TTACAGCCCTTCGGCGATGAT |  |  |  |  |

Note: PCR conditions: initial denaturation 94 °C 5 min; 30 cycles of 94 °C 45 sec, Ta 45 sec, 72 °C 1 min; final extension 72 °C 10 min. * - screening primers, † - sequencing primers. In *qnrB* and *qnrS*, screening primers were used first and various combinations of sequencing primers were subsequently utilized in order to obtain complete sequence of respective genes. In *bla*_CTX-M_, the isolates were first screened using universal primers PANCTX-M-F and PANCTX-M-R. The genes were afterwards classified into the group 1 or 4 using the screening primers. Complete sequences were utilized using sequencing primers for the respective *bla*_CTX-M_ group.
