# Supplementary material for: Characteristics of Quinolone Resistance in Escherichia coli Isolates from Humans, Animals, and the Environment in the Czech Republic
Source: Front Microbiol. 2017 Jan 9;7:2147. doi: 10.3389/fmicb.2016.02147 (PMC5220107; doi:10.3389/fmicb.2016.02147)
Supplement: Supplementary file 2 [file Table2.DOC]

**S2:** Percentage of resistant PMQR-positive isolates of *E. coli* to tested antimicrobial agents

| **Origin** | **No. of collected isolates** | **AMP** | **AMS** | **CZL** | **CRX** | **GEN** | **SXT** | **COL** | **OXO** | **OFL** | **TET** | **AZT** | **PIP** | **PPT** | **CPR** | **CTX** | **CTZ** | **CPM** | **CPS** | **MER** | **CIP** | **TIG** | **TOB** | **AMI** |
| --- | --- | --- | --- | --- | --- | --- | --- | --- | --- | --- | --- | --- | --- | --- | --- | --- | --- | --- | --- | --- | --- | --- | --- | --- |
| Hospital | 220 | 97,1 | 60 | 80 | 67,1 | 24,3 | 72,9 | 0 | 81,3 | 94,3 | 82,9 | 58,6 | 95,7 | 32,9 | 34,3 | 62,9 | 42,9 | 38,6 | 4,3 | 0 | 91,4 | 2,9 | 85,7 | 42,9 |
| Community | 83 | 92,9 | 57,1 | 64,3 | 28,6 | 28,6 | 64,2 | 0 | 92,9 | 85,7 | 92,9 | 50 | 92,9 | 28,6 | 42,9 | 50 | 42,9 | 50 | 7,1 | 0 | 85,7 | 0 | 64,3 | 50 |
| Chicken | 156 | 70,6 | 17,6 | 29,4 | 5,9 | 11,8 | 35,3 | 0 | 52,9 | 41,2 | 76,5 | 5,9 | 58,8 | 0 | 5,9 | 5,9 | 5,9 | 5,9 | 0 | 0 | 41,2 | 0 | 5,9 | 0 |
| Turkey | 105 | 96,1 | 35,3 | 15,7 | 0 | 3,9 | 19,6 | 0 | 33,3 | 31,4 | 86,3 | 0 | 90,2 | 0 | 0 | 0 | 0 | 0 | 0 | 0 | 25,5 | 0 | 3,9 | 0 |
| Rook | 114 | 75 | 20 | 20 | 20 | 0 | 40 | 0 | 30 | 25 | 75 | 20 | 70 | 5 | 20 | 20 | 5 | 0 | 0 | 0 | 25 | 0 | 0 | 0 |
| Wastewater | 372 | 78,9 | 10 | 14,4 | 5,6 | 14,4 | 58,9 | 0 | 27,8 | 34.4 | 83,3 | 10 | 77,8 | 1,1 | 4,4 | 6,7 | 6,7 | 6,7 | 0 | 0 | 27,8 | 0 | 26,7 | 1,1 |

Note: AMP - ampicillin; AMS - ampicillin/sulbactam; CZL - cefazoline; CRX - cefuroxime; GEN - gentamicin; SXT - co-trimoxazole; COL - colistin; OXO - oxolinic acid; OFL - ofloxacin; TET - tetracycline; AZT - aztreonam; PIP - piperacillin; PPT - piperacillin/tazobactam; CPR - cefoperazone; CTX - cefotaxime; CTZ - ceftazidime; CPM - cefepime; CPS - cefoperazone/sulbactam; MER - meropenem; CIP - ciprofloxacin; TIG - tigecycline; TOB - tobramycin; AMI – amikacin
