# Supplementary material for: Characteristics of Quinolone Resistance in Escherichia coli Isolates from Humans, Animals, and the Environment in the Czech Republic
Source: Front Microbiol. 2017 Jan 9;7:2147. doi: 10.3389/fmicb.2016.02147 (PMC5220107; doi:10.3389/fmicb.2016.02147)
Supplement: Supplementary file 3 [file Table3.DOC]

**S3 :** Diversity of single ST among *E. coli* isolates from different areas of collection

| **ST type / Origin** | **Hospital** | **Community** | **Chicken** | **Turkey** | **Rook** | **Wastewater** | **Total** |
| --- | --- | --- | --- | --- | --- | --- | --- |
| ST10 | - | - | 1 | 2 | 1 | 1 | 5 |
| ST23 | - | - | - | - | - | 3 | 3 |
| ST34 | - | - | - | - | - | 1 | 1 |
| ST43 | - | - | 1 | - | - | - | 1 |
| ST48 | - | - | 1 | 8 | - | 4 | 13 |
| ST58 | - | - | - | - | - | 2 | 2 |
| ST69 | - | 1 | - | - | - | - | 1 |
| ST88 | - | - | 1 | 2 | - | - | 3 |
| ST93 | 1 | - | - | - | - | - | 1 |
| ST95 | - | - | - | 9 | - | 1 | 10 |
| ST108 | - | - | - | 1 | - | - | 1 |
| ST117 | - | - | 1 | - | - | - | 1 |
| ST131 | 21 | 4 | - | - | - | 1 | 26 |
| ST155 | - | - | - | 3 | - | - | 3 |
| ST156 | - | - | - | - | - | 1 | 1 |
| ST162 | - | - | - | 4 | - | - | 4 |
| ST167 | 1 | - | - | - | - | - | 1 |
| ST224 | 1 | - | - | - | - | - | 1 |
| ST226 | - | - | 1 | 1 | - | - | 2 |
| ST278 | - | - | - | - | 1 | - | 1 |
| ST297 | - | - | - | - | 2 | - | 2 |
| ST355 | - | - | - | 19 | - | - | 19 |
| ST393 | 1 | - | - | - | - | - | 1 |
| ST405 | 2 | 1 | - | - | - | - | 3 |
| ST410 | 2 | - | - | - | - | - | 2 |
| ST428 | 1 | - | - | - | - | 1 | 2 |
| ST442 | - | - | - | - | - | 1 | 1 |
| ST453 |  | 2 | - | - | - | - | 2 |
| ST533 | - | - | 1 | - | 1 | - | 2 |
| ST540 | - | - | - | - | - | 1 | 1 |
| ST542 | - | - | - | - | - | 1 | 1 |
| ST 617 | 2 | - | - | - | - | 1 | 3 |
| ST641 | - | - | - | - | - | 3 | 3 |
| ST648 | - | - | - | - | 1 | - | 1 |
| ST752 | - | - | 1 | - | - | - | 1 |
| ST774 | - | - | - | - | 1 | - | 1 |
| ST776 | - | - | - | - | - | 1 | 1 |
| ST1139 | - | - | - | - | - | 3 | 3 |
| ST1611 | - | - | - | - | - | 1 | 1 |
| ST1722 | 1 | - | - | - | - | - | 1 |
| ST 2011 | - | - | - | - | 1 | - | 1 |
| ST2207 | - | - | - | 1 | - | - | 1 |
| ST2497 | - | - | 1 | - | - | - | 1 |
| ST3107 | - | - | - | - | 1 | - | 1 |
| ST3331 | - | - | - | - | - | 1 | 1 |
| ST3714 | - | - | - | - | 1 | - | 1 |
| ST3721 | - | 1 | - | - | - | - | 1 |
| ST4558 | - | - | - | - | - | 1 | 1 |
| **Total** | 33 | 9 | 9 | 50 | 10 | 29 | **140** |
